# Role of Damage Control Surgery in Severe Liver Trauma: A Systematic Review of Mortality, Morbidity, and ICU Stay

**DOI:** 10.7759/cureus.100222

**Published:** 2025-12-27

**Authors:** Beshr Mosa Basha, Ammar M Eskander, Rana M Haris, Ahmad Hamdan, Shashwat Shetty, Yousif Osman, Aliaa Alkhazendar, Ifrah Saleem

**Affiliations:** 1 General Surgery, Dr Sulaiman Al-Habib Medical Group, Al Khobar, SAU; 2 General Surgery, Doctors Hospital and Medical Center, Lahore, PAK; 3 Orthopaedics, Hillingdon Hospital, Uxbridge, GBR; 4 General Surgery, National Ribat University Hospital, Khartoum, SDN; 5 Medicine, The Islamic University of Gaza, Gaza, PSE; 6 General Practice, Dow University of Health Sciences, Civil Hospital Karachi, Karachi, PAK

**Keywords:** damage control surgery, hepatic injury, mortality, severe liver trauma, trauma surgery

## Abstract

This systematic review evaluates the role of damage control surgery (DCS) in managing severe hepatic trauma (AAST grade III-V), focusing on mortality, morbidity, and ICU stay. A comprehensive search of PubMed, Embase, Scopus, and the Cochrane Library identified six studies with a combined sample size of 1,497 patients, analyzed according to Preferred Reporting Items for Systematic Reviews and Meta-Analyses (PRISMA) 2020 guidelines. DCS comprising perihepatic packing, temporary abdominal closure, and staged re-laparotomy was shown to improve short-term survival by rapidly controlling hemorrhage and stabilizing physiology in critically unstable patients. Evidence indicated that early re-laparotomy (within 24-48 hours) reduced ICU stay and postoperative complications compared with delayed re-exploration. Despite improved outcomes, mortality remained influenced by initial physiological instability and associated injuries. Most studies presented a moderate risk of bias due to retrospective designs and heterogeneity in protocols. Overall, DCS remains a cornerstone in the management of severe liver trauma, and future multicenter prospective studies are needed to refine operative timing, standardize protocols, and optimize patient outcomes.

## Introduction and background

Severe hepatic trauma continues to pose one of the most formidable challenges in trauma surgery. Patients who present with unstable physiology, characterized by profound hemorrhage, acidosis, hypothermia, and coagulopathy, historically face mortality rates exceeding 50% in unstable cases [1]. In such situations, attempts at definitive repair during the initial laparotomy often lead to worsening physiological derangement and higher mortality. The evolution of damage control surgery (DCS) revolutionized trauma care by emphasizing rapid hemorrhage control, temporary stabilization, and staged definitive repair rather than prolonged and complex interventions in critically ill patients [2]. In the context of liver trauma, DCS protocols typically include perihepatic packing, temporary abdominal closure, and delayed re-laparotomy once physiological homeostasis has been restored [3]. This approach is particularly valuable in high-grade hepatic injuries according to the American Association for the Surgery of Trauma Liver Injury Scale (AAST grade III-V), where extensive bleeding and associated shock can rapidly lead to the lethal triad of coagulopathy, hypothermia, and acidosis. Early hemostatic control through packing, the Pringle maneuver, and selective vascular occlusion provides time for intensive care resuscitation, correction of metabolic derangements, and optimization before definitive hepatic repair [4].

Despite the reduction in early mortality attributed to DCS, important clinical questions remain regarding its long-term effects on morbidity, ICU stay, and overall outcomes. Some studies have suggested that delayed re-laparotomy after DCS is associated with increased postoperative complications and prolonged ICU stay, while early re-intervention within 24-48 hours can improve survival and reduce morbidity [5]. However, significant variability in DCS indications, operative techniques, and timing across institutions complicates outcome comparison. Furthermore, integration of damage control resuscitation (DCR) principles, including balanced transfusion ratios, permissive hypotension, and restricted crystalloid use, has refined the modern application of DCS but requires ongoing evaluation [6].

The primary aim of this review is to systematically evaluate the existing evidence on the role of DCS in the management of severe hepatic trauma (AAST grade III-V), with a particular focus on its impact on key clinical outcomes, including mortality, morbidity, and length of ICU stay. By analyzing data from published studies, this review seeks to determine the effectiveness of DCS as a life-saving strategy in patients presenting with hemodynamic instability and complex liver injuries. The secondary aim is to explore how the timing of re-laparotomy, specifically early versus delayed re-exploration, and the incorporation of modern resuscitative strategies such as balanced transfusion ratios, permissive hypotension, and correction of coagulopathy influence postoperative outcomes. Additionally, this review aims to identify gaps in the current literature regarding patient selection criteria, standardization of DCS protocols, and long-term functional recovery, emphasizing the need for prospective multicenter studies to guide future clinical practice and improve outcomes in severe hepatic trauma.

## Review

Materials and methods

Search Strategy

A comprehensive literature search was conducted according to the Preferred Reporting Items for Systematic Reviews and Meta-Analyses (PRISMA) 2020 guidelines [7]. The databases PubMed, Embase, Scopus, and the Cochrane Library were systematically searched for studies published up to October 2025 evaluating the role of DCS in severe hepatic trauma. Search terms included “damage control surgery,” “liver trauma,” “hepatic injury,” “mortality,” “morbidity,” and “ICU stay.” Boolean operators AND and OR were applied to combine and refine keywords. Reference lists of relevant studies were also manually reviewed to identify additional eligible articles. Only English-language human studies with full-text availability were included.

Eligibility Criteria

The eligibility criteria for this systematic review were structured using the PICO framework [8]. Population (P) included adult human patients (≥18 years) diagnosed with severe hepatic trauma (AAST grade III-V). Intervention (I) was the application of DCS, including perihepatic packing, temporary abdominal closure, and planned re-laparotomy. Comparator (C) included early versus delayed re-laparotomy or other definitive hepatic repair techniques. Outcomes (O) focused on mortality, morbidity, and ICU stay, along with postoperative complications and physiological recovery. Exclusion criteria included case reports, animal studies, editorials, and conference abstracts to ensure methodological rigor and relevance. Only peer-reviewed full-text human studies published in English were included.

Study Selection

All identified studies were independently screened by two reviewers following the PRISMA 2020 guidelines. Titles and abstracts were initially evaluated to exclude duplicates and studies irrelevant to DCS in severe hepatic trauma. Full-text articles of potentially eligible studies were then assessed in detail against the predefined inclusion and exclusion criteria. Any disagreements between reviewers were resolved through discussion, and when necessary, consultation with a third senior reviewer ensured objectivity and consensus. Studies that met all eligibility requirements were included in the final qualitative synthesis and data extraction phase.

Data Extraction

Data were independently extracted from all included studies using a standardized data extraction form developed for this review. Two reviewers systematically collected data to ensure accuracy and minimize selection bias. Extracted variables included study characteristics (author, year, design, and country), patient demographics, injury severity (AAST grade III-V), intervention details (type of DCS, timing of re-laparotomy), and comparator groups where applicable. Key clinical outcomes such as mortality, morbidity, ICU stay, and postoperative complications were also recorded. This structured approach ensured a consistent and comprehensive synthesis of relevant data across all included studies.

Risk of Bias Assessment

The risk of bias for each included study was assessed using validated tools appropriate to the study design. As all included studies were observational (prospective or retrospective cohort studies), the ROBINS-I tool (Risk of Bias in Non-randomized Studies of Interventions) was applied to evaluate methodological quality [9]. Each study was assessed across key domains, including selection bias, confounding, measurement of outcomes, completeness of data, and selective reporting. Studies were then classified as having low, moderate, or high risk of bias, with justifications provided based on study design, sample size, and internal validity. Most studies were rated as having a moderate risk of bias due to retrospective design and heterogeneity in DCS protocols, though all provided clearly defined outcomes and adequate follow-up.

Data Synthesis

Due to heterogeneity in study design, patient populations, DCS protocols, and outcome measures, a narrative synthesis was performed instead of a quantitative meta-analysis. The synthesis focused on summarizing the impact of DCS on key clinical outcomes, including mortality, morbidity, and ICU stay, among patients with severe hepatic trauma (AAST grade III-V). Comparisons were made between early and delayed re-laparotomy, emphasizing their effects on postoperative recovery and complications. The findings were integrated to highlight common trends, pathophysiological implications, and areas of variability across studies, providing insight into how DCS timing and resuscitative strategies influence clinical outcomes and guide trauma management decisions.

Results

Study Selection Process

**Figure 1:**
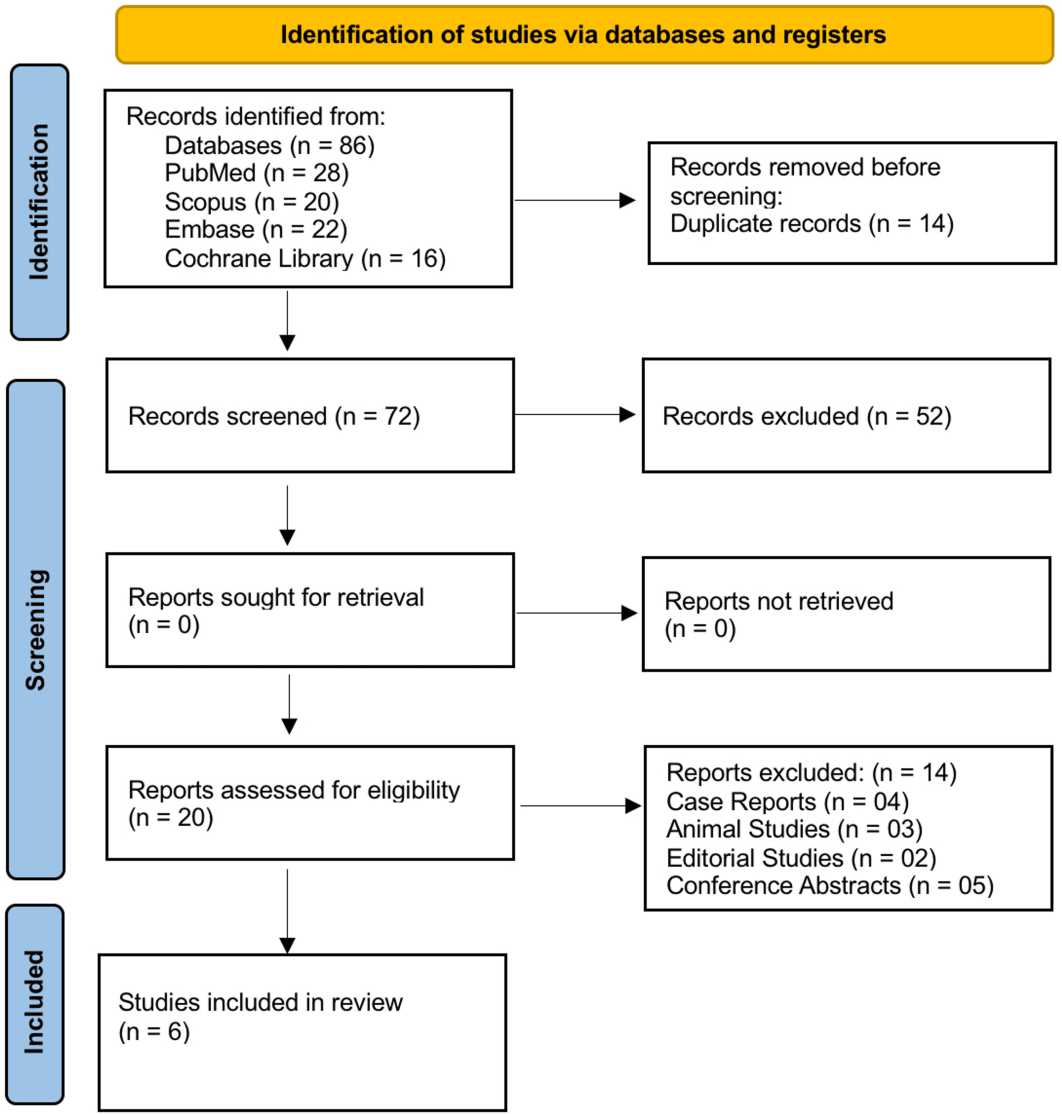
PRISMA 2020 flow diagram PRISMA: Preferred Reporting Items for Systematic Reviews and Meta-Analyses.

Figure 1 illustrates the study selection process conducted in accordance with the PRISMA 2020 guidelines. A total of 86 records were identified through comprehensive searches of four electronic databases: PubMed (n = 28), Embase (n = 22), Scopus (n = 20), and the Cochrane Library (n = 16). After the removal of 14 duplicate records, 72 studies remained for initial screening based on titles and abstracts. Of these, 52 studies were excluded, as they did not meet the predefined inclusion criteria, primarily due to irrelevant focus or insufficient outcome data. The remaining 20 full-text articles were assessed for eligibility. Upon detailed review, 14 articles were excluded, including 4 case reports, 3 animal studies, 2 editorials, and 5 conference abstracts. No studies were excluded due to unavailability of full text. After applying all inclusion and exclusion criteria, a total of 6 studies were included in the final qualitative synthesis for this systematic review.

Characteristics of the Selected Studies

Table 1 summarizes the characteristics of the six studies included in this systematic review. Kang et al. (2020) investigated 65 patients with high-grade liver injuries managed by DCS with perihepatic packing and found that early re-laparotomy (≤48 hours) significantly reduced ICU stay and postoperative pneumonia compared with delayed re-exploration [10]. Hosseinpour et al. (2023) analyzed 914 patients with severe hepatic trauma (AAST ≥ III), showing that delayed hepatic resection during DCS was associated with prolonged ICU stay and higher complication rates, though mortality differences were not significant [11]. Giannoudis et al. (2015) evaluated 121 trauma patients and reported improved hemodynamic stabilization with DCS, though mortality reached 33.1% in those with hemorrhagic shock and ARDS [12]. Søreide et al. (2009) compared early DCS with non-operative management and definitive repair in 144 patients, noting an overall mortality of 15%, primarily in those presenting with shock and coagulopathy [13]. Leonardi et al. (2023) studied 62 patients undergoing DCS for abdominal trauma, identifying a high mortality rate (59.6%) due to multi-organ failure and sepsis [5]. Finally, Silva et al. analyzed 191 trauma patients, including 21 who underwent DCS, and found a mean ICU stay of 14.38 ± 11.63 days and a mortality rate of 22.7%, with infections and ARDS being the most frequent complications. Collectively, these studies demonstrate that early re-laparotomy following DCS improves short-term outcomes, though mortality remains influenced by initial physiological instability and injury severity.

**Table 1:** Characteristics of the Selected Studies AAST: American Association for the Surgery of Trauma, DCS: damage control surgery, ICU: intensive care unit, ARDS: acute respiratory distress syndrome.

Authors and Year	Population (P)	Exposure/Condition (I)	Comparator (C)	Outcomes (O)	Pathophysiological Findings	Anatomical Impact	Damage Control Surgery	ICU Stay	Mortality	Morbidity
Kang et al., 2020 [10]	65 high-grade liver injury patients	DCS + perihepatic packing + early re-laparotomy (≤48h)	DCS + perihepatic packing + delayed (>48h) re-laparotomy	ICU stay, mortality, pneumonia	Hemodynamic instability, coagulopathy, bleeding	AAST grade III–V liver laceration	Yes	9 (5–31) vs 18 (11–28) days	16.7% vs 14.6%	Pneumonia lower in the ≤48 h group
Hosseinpour et al., 2023 [11]	914 adult trauma patients (AAST ≥ III)	DCS + hepatic resection (early)	DCS + hepatic resection (delayed)	ICU stay, mortality, complications	Shock, acidosis, coagulopathy	Severe liver injury (AAST ≥ III) ± other organ injuries	Yes	ICU LOS increased with delayed resection (β ICU +0.198)	No significant difference	Higher complications in delayed resection (aOR 1.842)
Giannoudis et al., 2015 [12]	121 trauma patients	DCS: liver packing + planned re-laparotomy	Definitive hepatic repair in initial surgery	ICU stay, mortality, complications	Hemorrhagic shock, coagulopathy, ARDS in non-survivors	AAST grade III–V liver laceration	Yes	Mean/median ICU stay recorded in the study	33.1%	Bleeding, ARDS, infections
Søreide et al., 2009 [13]	144 patients with severe liver injuries	Early laparotomy/DCS	Non-operative management or early definitive repair	Mortality, criteria for management selection	Shock on admission, coagulopathy	AAST grade III–V liver injury ± splenic/head injuries	Yes	Not specified	15% overall	Shock-related complications
Leonardi et al., 2023 [5]	62 patients undergoing DCS for abdominal trauma (liver included)	DCS	–	Mortality, predictive factors	Hypotension, acidosis, coagulopathy	Liver + other abdominal organs	Yes	Not specified	59.6%	Multi-organ failure, sepsis
Silva et al., 2017 [14]	191 multiple trauma patients	Surgical management, including DCS (21 patients)	Other surgical techniques (hepatorrhaphy etc.)	ICU stay, mortality, hospitalization time	Severe bleeding, shock, transfusion requirement	Liver injury AAST III–V	Yes	14.38 ± 11.63 days (mean)	22.7%	Infections, bleeding, ARDS

Risk of Bias Assessment

All six studies included in this review were assessed for methodological quality using the ROBINS-I tool (Risk of Bias in Non-Randomized Studies of Interventions). Overall, most studies demonstrated a moderate risk of bias, primarily due to their retrospective design and heterogeneity in DCS protocols. Kang et al. (2020) and Hosseinpour et al. (2023) were rated as moderate risk, given potential selection and confounding biases, despite clear outcome definitions and adequate follow-up [10,11]. Giannoudis et al. (2015) showed low to moderate risk due to its prospective design and standardized protocol, though generalizability was limited to a single center [12]. Søreide et al. (2009) [13] and Leonardi et al. (2023) [5] carried moderate to high risk owing to incomplete data and small, heterogeneous samples, while Silva et al. (2017) was rated as having a moderate risk due to its retrospective design but provided clear and reliable outcome reporting, as shown in Figure 2 [14].

**Table 2:** Risk of Bias Assessment ROBINS-I: Risk of Bias in Non-randomized Studies of Interventions, DCS: damage control surgery, ICU: intensive care unit.

Study	Study Design	Risk of Bias Tool	Risk of Bias Rating	Justification
Kang et al., 2020 [10]	Retrospective cohort	ROBINS-I (Risk Of Bias In Non-randomized Studies - of Interventions)	Moderate	Retrospective design, small sample, possible selection bias, but outcomes clearly defined and follow-up adequate
Hosseinpour et al., 2023 [11]	Retrospective cohort/registry analysis	ROBINS-I	Moderate	Large sample, multicenter, but retrospective, with possible unmeasured confounding and variability in DCS protocols
Giannoudis et al., 2015 [12]	Prospective cohort (single center)	ROBINS-I	Low to Moderate	Prospective design, standardized DCS protocol, but a single center limits generalizability
Søreide et al., 2009 [13]	Retrospective cohort	ROBINS-I	Moderate to High	Retrospective, mixed management strategies, ICU/complication data incomplete in the abstract
Leonardi et al., 2023 [5]	Retrospective cohort	ROBINS-I	Moderate to High	Retrospective, small sample, trauma heterogeneity (liver + other organs), outcomes influenced by multiple confounders
Silva et al., 2017 [14]	Retrospective cohort	ROBINS-I	Moderate	Retrospective design, variability in reporting and management, but clear outcome measures and reasonable data completeness.

Discussion

This systematic review highlights the essential role of DCS in managing severe hepatic trauma, particularly in patients presenting with hemodynamic instability and physiological compromise. DCS has proven to be a life-saving intervention by prioritizing rapid hemorrhage control, contamination prevention, and stabilization before definitive repair. In patients with AAST grade III-V liver injuries, this staged surgical approach interrupts the lethal triad of acidosis, coagulopathy, and hypothermia, improving short-term survival outcomes. The timing of re-laparotomy emerged as a critical factor influencing recovery. Studies consistently showed that early re-laparotomy (within 24-48 hours) following initial DCS was associated with shorter ICU stay and fewer complications, while delayed re-exploration correlated with higher rates of infection, sepsis, and multi-organ failure [10,11]. Early intervention allows for timely removal of perihepatic packs and correction of residual bleeding once physiological parameters are optimized.

However, the available evidence remains limited by methodological variability. Most studies were retrospective, single-center, and demonstrated heterogeneity in DCS protocols, resuscitation strategies, and definitions of “early” versus “delayed” intervention. These limitations, along with a moderate to high risk of bias, restrict the generalizability of findings. Additionally, long-term outcomes, such as hepatic function recovery and late complications, were rarely reported, underscoring a need for more robust data. Future research should focus on standardizing DCS and DCR protocols, integrating modern strategies such as balanced transfusion ratios and early correction of coagulopathy. Prospective multicenter studies and trauma registries are crucial to refine surgical timing and optimize care pathways. In summary, DCS remains a cornerstone in the management of severe liver trauma, but further evidence-based standardization is needed to improve morbidity, reduce ICU burden, and enhance survival outcomes worldwide.

## Conclusions

This systematic review concludes that DCS plays a crucial and life-saving role in the management of severe hepatic trauma (AAST grade III-V) by prioritizing rapid hemorrhage control, physiological stabilization, and staged definitive repair. The evidence consistently demonstrates that DCS significantly reduces early mortality in hemodynamically unstable patients and that early re-laparotomy (within 24-48 hours) is associated with shorter ICU stays and fewer postoperative complications compared with delayed re-exploration. Despite variations in protocols and study design, DCS remains the cornerstone of surgical management for complex liver injuries, effectively interrupting the lethal triad of acidosis, coagulopathy, and hypothermia. Future research should aim to standardize DCS protocols, integrate DCR strategies, and establish multicenter registries to better define optimal timing and improve outcomes related to mortality, morbidity, and ICU stay in patients with severe liver trauma.
